# Compromise or optimize? The breakpoint anti-median

**DOI:** 10.1186/s12859-016-1340-y

**Published:** 2016-12-15

**Authors:** Caroline Anne Larlee, Alex Brandts, David Sankoff

**Affiliations:** Department of Mathematics and Statistics, University of Ottawa, 585 King Edward Avenue, Ottawa, K1N 6N5 Canada

**Keywords:** Median problem, Gene order, Breakpoint distance, Gene adjacency

## Abstract

**Background:**

The median of *k*≥3 genomes was originally defined to find a compromise genome indicative of a common ancestor. However, in gene order comparisons, the usual definitions based on minimizing the sum of distances to the input genomes lead to degenerate medians reflecting only one of the input genomes. “Near-medians”, consisting of equal samples of gene adjacencies from all the input genomes, were designed to restore the idea of compromise to the median problem.

**Result:**

We explore adjacency sampling constructions in full generality in the case *k*=3, with given overlapping sets of adjacencies in the three genomes, where all adjacencies in two-way or three-way overlaps are included in the sample. We require the construction to be maximal, in the sense that no additional proportion of adjacencies from any of the genomes may be added without violating the local linearity of the genome. We discover that in incorporating as many adjacencies as possible, evenly from all the input genomes, we are actually maximizing, rather than minimizing, the sum of distances over all other maximal sampling schemes.

**Conclusions:**

We propose to explore compromise instead of parsimony as the organizing principle for the small phylogeny problem.

## Background

In comparative genomics, a median genome *m* for a set of *k*≥3 given genomes *g*_1_,…,*g*_*k*_ in a metric space (*G*,*d*) minimizes

1 $$ S(m)=\sum_{i=1}^{k} d(m,g_{i})  $$

over all *m*∈*G* [1]. This is meant to embody a compromise among the given genomes, usually as an inference of a common ancestor.

While the simplicity of the median concept is appealing, and it has stimulated a large literature [2], it suffers from important shortcomings: it is hard to calculate [3–5] for almost all (*G*,*d*), and is not a compromise in the most important contexts. For example, for *k*≥3 random signed permutations of length *n*, and for *d* the “breakpoint distance”, the median tends to one or more of the given permutations as *n* increases [6–8].

**Fig. 1:**
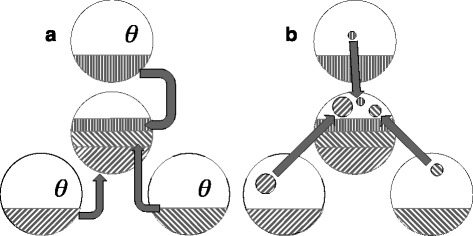
**a** First sampling of *θ* *n* adjacencies from each of three genomes. **b** Supplementary sampling of residual adjacencies consisting of two free ends

The “near median” was proposed to get around these difficulties [9]. For *k* random genomes, the same proportion of gene adjacencies is sampled from each one, in such a way that the union of the samples is compatible – an “end” of a gene is adjacent to no more than one other gene end. The proportion of the compromise genome remaining to be constructed can be filled by any matching of the unassembled gene ends, as in Fig. 1.

If comparable proportions of the constructed genome are contributed by each of the *k* genomes, the spirit of compromise is ensured. The sampling is rapidly carried out.

In the original paper [9], only the following, highly symmetrical cases were studied for *k*=3: three purely random genomes, three genomes all with common adjacencies forming a proportion *ψ* of their adjacencies, and three genomes all with a proportion *ψ* of common adjacencies and additional proportions *ω*_1,2_,*ω*_1,3_,*ω*_2,3_ of adjacencies in their pairwise intersections. We only investigated the maximum *θ* such that the same proportion *θ* could be sampled from the three input genomes.

In the present paper we extend our analysis to examine the entire set of compatible triples (*θ*_1_,*θ*_2_,*θ*_3_).

In the process, we discover the surprising fact that not only does our sampling procedure fail to minimize the sum in (1), it actually maximizes it! In doing so, it illustrates that the search for optimality and compromise are at cross-purposes. In concluding we suggest how the goal of compromise may be used as a criterion for the small phylogeny problem in the place of optimality.

## Results

### Definitions

Consider three signed genomes, *g*_1_,*g*_2_ and *g*_3_, each consisting of one or more chromosomes – circular orderings – containing the same *n* genes and each containing *n* gene adjacencies. Although we assume the chromosomes are circular for technical simplicity, the analysis is essentially the same for linear, circular, unichromosomal or multichromosomal genomes; the effect of allowing a bounded number >1 of chromosomes would be $\mathcal {O}$ (*n*) as would be the differences between circular and linear models. We also assume *n* is large so that for an arbitrary proportion *θ*, the $\mathcal {O}$ (1) difference between *θ* *n* and the nearest integer to *θ* *n* may be neglected. The probabilistic justification behind these assumptions is discussed in [9].

That the genomes are “signed” means the genes have polarity, so the two ends of a gene have distinct labels. Each adjacency is thus an unordered pair of the 2*n* gene ends, chosen from among ${2n \choose 2}$ possibilities. For a genome to be “compatible”, no gene end may be part of more than one adjacency. There is no constraint involving the two ends of the same gene, other than that both ends of all genes must eventually be included in any genome we construct. E.g., there is no constraint against the two ends of the same gene being adjacent, forming a minimal circular chromosome.

We are given that *g*_1_,*g*_2_ and *g*_3_ have a proportion *ψ* of common adjacencies and proportions *ω*_1,2_,*ω*_1,3_,*ω*_2,3_ of adjacencies in their pairwise intersections.

The breakpoint distance between two genomes can be defined as *d*=*n*−*a*, where *a* is the number of adjacencies they contain in common. For example *d*(*g*_1_,*g*_3_)=*n*−*ψ*−*n* *ω*_1,3_.

For a genome *x* the sum of the normalized distances to the three input genomes,

2 $$ s(x)=\frac{1}{n}\sum_{i=1}^{3} d(x,g_{i}),  $$

is called its score.

A sample is defined by a triple of (*θ*_1_,*θ*_2_,*θ*_3_) each between 0 and 1 and summing to less than 1−*ψ*−*ω*_1,2_−*ω*_1,3_−*ω*_2,3_ such that a random choice of *θ*_1_ *n* adjacencies from *g*_1_,*n* *θ*_2_ from *g*_2_, and *n* *θ*_3_ from *g*_3_ are compatible with each other and with the adjacencies in the overlaps. A sample is “randomly completed” to form a genome with *n* genes by the addition of 1−*ψ*−*ω*_1,2_−*ω*_1,3_−*ω*_2,3_ adjacencies constructed by randomly pairing gene ends that are not in any of the adjacencies in the sample or in the overlaps. In other words, to focus on the purely statistical consequences of the sampling procedure we thus do not consider the increment in the number of adjacencies obtainable in individual instances by the ad hoc matching algorithms developed in [9]. The random completion process does not add to the number of adjacencies in the sample in common with one, two or three of *g*_1_,*g*_2_ and *g*_3_.

A “maximal” sample is one where none of the *θ*_*i*_ may be increased without causing a number (greater than $\mathcal {O}$ (*n*)) of incompatible adjacencies.

### The construction

From the three input genomes, we construct a set containing adjacencies sampled in various proportions among *g*_1_,*g*_2_ and *g*_3_ and including the adjacencies in the given two-way and three-way overlaps, randomly completed by pairs of gene ends matched from among the remaining unsampled ends. The only constraint in adding an adjacency is that it must have two “free ends”; i.e., no adjacency previously included, whether given or sampled, may contain either of these two ends.

Note that two random permutations can be expected to have virtually no adjacencies in common; the expectation of the number of adjacencies goes to a small constant as *n* increases [10].

As an illustration, consider the case where *ψ*=*ω*_1,2_=*ω*_1,3_=*ω*_2,3_=0. As a first step, we may select *θ*_1_ *n* adjacencies from *g*_1_, where 0≤*θ*_1_≤1. Then for *g*_2_, the expected proportion of “two free ends”, adjacencies where neither end appears in a previously selected *g*_1_ adjacency, is (1−*θ*_1_)^2^. As long as *θ*_1_≠1, we can pick *θ*_2_ *n* adjacencies from genome *g*_2_ that do not conflict with any of those selected from *g*_1_, where 0≤*θ*_2_≤(1−*θ*_1_)^2^.

Similarly, having then selected *θ*_1_ *n* pairs of gene ends from *g*_1_ and *θ*_2_ *n* pairs of gene ends from *g*_2_, the expected proportion of pairs in *g*_3_ with two free ends is (1−*θ*_1_−*θ*_2_)^2^. As long as this quantity is greater than zero, we can chose some *θ*_3_ *n* compatible pairs from *g*_3_.

For a maximal sample we should take the maximum number of pairs from *g*_3_, i.e., the maximum *θ*_3_, given *θ*_1_ and *θ*_2_, i.e.,

3 $$ \theta_{3}=(1-\theta_{1}-\theta_{2})^{2}.  $$

Adding the remainder of the gene ends not in any adjacency in *g*_1_,*g*_2_ or *g*_3_ using any matching to form pairs, we obtain a genome *x*, and Eqs. (2) and (3) give

4 $$ s(x)= 3-(\theta_{1}+\theta_{2}+(1-2\theta_{1}-2\theta_{2}+ 2\theta_{1}\theta_{2}+{\theta_{1}^{2}}+{\theta_{2}^{2}})).  $$

**Fig. 2:**
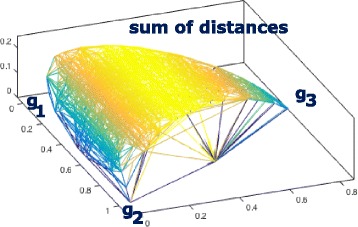
Surface of distance sum as a function of *θ*_1_,*θ*_2_,*θ*_3_, in barycentric coordinates when *θ*_3_ is maximized as a function of *θ*_1_ and *θ*_2_

Figure 2 depicts a surface described by the values of *s*(*x*) of the vertices in a Delaunay triangulation of (*θ*_1_,*θ*_2_,*θ*_3_) in barycentric coordinates [11, 12]. It appears from this depiction that “compromise” values of (*θ*_1_,*θ*_2_,*θ*_3_), i.e., around the interior of the triangle, give the largest values, not the smallest, value of *s*(*x*).

Indeed, the derivative of the expression in (4) with respect to either *θ*_1_ or *θ*_2_,

5 $$ s'(x)=1-2\theta_{1}-2\theta_{2},  $$

is zero iff *θ*_1_+*θ*_2_=0.5. The second derivatives are negative, so the surface is convex.

Examining some values of max*θ*_3_ and *s*(*x*) in Table 1, we confirm that the maximum value of *s*(*x*) occur for a genome *x* where *θ*_1_+*θ*_2_=0.5 and *θ*_3_=0.25.

**Table 1:** Maximizing *θ*_3_ for various combinations of *θ*_1_ and *θ*_2_

*θ*_1_	*θ*_2_	max*θ*_3_	*s*
0.15	0.15	0.4900	2.2100
	0.20	0.4225	2.2275
	0.25	0.3600	2.2400
	0.30	0.3025	2.2475
	0.35	0.2500	2.2500
0.20	0.15	0.4225	2.2275
	0.20	0.3600	2.2400
	0.25	0.3025	2.2475
	0.30	0.2500	2.2500
	0.35	0.2025	2.2475
0.25	0.15	0.3600	2.2400
	0.20	0.3025	2.2475
	0.25	0.2500	2.2500
	0.30	0.2025	2.2475
	0.35	0.1600	2.2400
0.30	0.15	0.3025	2.2475
	0.20	0.2500	2.2500
	0.25	0.2025	2.2475
	0.30	0.1600	2.2400
	0.35	0.1225	2.2275
0.35	0.15	0.2500	2.2500
	0.20	0.2025	2.2475
	0.25	0.1600	2.2400
	0.30	0.1225	2.2275
	0.35	0.0900	2.2100

By symmetry, we can obtain all of:

6 $$\begin{array}{*{20}l} \theta_{1}+\theta_{2}=0.5\ \\ \theta_{2}+\theta_{3}=0.5\  \\ \theta_{3}+\theta_{1}=0.5 . \end{array} $$

The unique solution of all three equations is *θ*_1_=*θ*_2_=*θ*_3_=0.25.

**Fig. 3:**
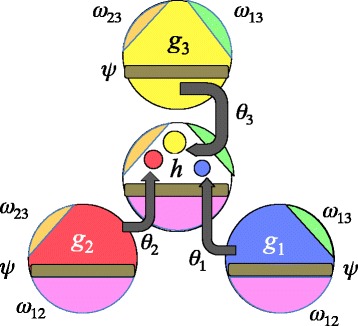
Sampling scheme showing variable proportions *θ*_1_,*θ*_2_,*θ*_3_, and given two-way intersections *ω*_1_,*ω*_2_,*ω*_3_ and three-way intersection *ψ*. All these contributions lower *s*. *White area* in genome *h* represent the randomly completed portion

Turning to the more general case where *ψ* and the *ω*_*i*,*j*_ are not required to be zero, as illustrated in Fig. 3, Eq. (4) becomes

7 $$ s(x)=3-(\theta_{1}+\theta_{2}+ \max\theta_{3}+\sum_{i\neq j}^{3}\omega_{i,j}+\psi).  $$

and Eq. (6) become

8 $$\begin{array}{*{20}l} \theta_{1}+\theta_{2}=0.5- \omega_{1,2}-\omega_{2,3}-\omega_{1,3}-\psi \\ \theta_{2}+\theta_{3}=0.5- \omega_{1,2}-\omega_{2,3}-\omega_{1,3}-\psi \  \\ \theta_{3}+\theta_{1}=0.5 - \omega_{1,2}-\omega_{2,3}-\omega_{1,3}-\psi \ . \end{array} $$

The unique solution of all three equations is

9 $$ \theta_{1}=\theta_{2}=\theta_{3}=0.25-0.5(\omega_{1,2}+\omega_{2,3}+\omega_{1,3}+\psi)  $$

which maximizes *s*(*x*) over all maximal samples.

We might imagine that it would be “fairer” to distribute adjacencies among the *θ*’s in the proportions:

10 $$ \begin{aligned} \theta_{1}:\theta_{2}:\theta_{3} & = \frac{1}{2}(\omega_{1,2}+\omega_{1,3})+\frac{\psi}{3}:\frac{1}{2}(\omega_{1,2}+\omega_{2,3}) \\ & \quad + \frac{\psi}{3}:\frac{1}{2}(\omega_{1,3}+\omega_{2,3})+\frac{\psi}{3}, \end{aligned}  $$

where each genome would contribute a number of adjacencies in proportion to the number it has already contributed in *ψ* and the *ω*’s. However, this is not a solution for the equations in (8) for general values of *ω*_1,2_,*ω*_1,3_ and *ω*_2,3_, and upon reflection, there is no reason to consider this a better compromise than an equal division of adjacencies among the three genomes, beyond the unbalances already inherent in the pairwise overlaps.

## Discussion

The breakpoint median minimizes the sum of the breakpoint distance to three given genomes but in doing so foregoes any property of “compromise” among the three, despite this being the original motivation for the median. The anti-median represents a complete emphasis on “compromise” instead of on shortest distances. Somewhat surprisingly, the anti-median actually maximizes the sum of the breakpoint distance to three given genomes, in the process assuring that none of the three input genomes is disproportionately represented, other than through its given overlap with the other two genomes.

Note that the anti-median genomes are constructed to have precise normalized distances from *g*_1_,*g*_2_, and *g*_3_, in the sense of their limiting behaviour as *n*→*∞*. This behaviour is predicated on the inclusion of all the adjacencies in the two-way and three-way overlap, and the completion of the sampled genome by random matching of unpaired ends. These anti-medians contrast with arbitrary random genomes whose normalized sums of scores to *g*_1_,*g*_2_, and *g*_3_ approach 3. At the other extreme, they also contrast with the “near medians” [9] completed by maximum matching algorithms, whose scores are less than those of the randomly completed samples constructed here.

## Conclusions

Median constructions form the basis of the steinerization strategy for solving the small phylogeny problem, finding the ancestral genomes to populate the ancestral nodes of a given phylogeny when the genomes at the leaf nodes are known. Each ancestral node in turn is subjected to a median search, based on its three neighbors, and this is iterated until convergence. This constitutes a search for a most parsimonious solution. But if we wish ancestral nodes to reflect all three neighboring nodes (in a binary tree), there is no obstacle in using anti-medians instead of medians, and actually searching for a *least* parsimonious solution, so that compromise becomes the organizing principle in the reconstruction. Exploring this becomes the most important project for future work on this subject.
